# Intrinsic Network Brain Dysfunction Correlates With Temporal Complexity in Generalized Anxiety Disorder and Panic Disorder

**DOI:** 10.3389/fnhum.2021.647518

**Published:** 2021-07-15

**Authors:** Li Xu, Huazhen Xu, Huachen Ding, Jinyang Li, Chun Wang

**Affiliations:** ^1^Nanjing Brain Hospital Affiliated to Nanjing Medical University, Nanjing, China; ^2^School of Psychology, Nanjing Normal University, Nanjing, China; ^3^Cognitive Behavioral Therapy Institute of Nanjing Medical University, Nanjing, China; ^4^Functional Brain Imaging Institute of Nanjing Medical University, Nanjing, China

**Keywords:** generalized anxiety disorder, panic disorder, dynamic functional connectivity, neural networks, functional MRI

## Abstract

**Background:** Generalized anxiety disorder (GAD) and panic disorder (PD) are the two severe subtypes of anxiety disorders (ADs), which are similar in clinical manifestation, pathogenesis, and treatment. Earlier studies have taken a whole-brain perspective on GAD and PD in the assumption that intrinsic fluctuations are static throughout the entire scan. However, it has recently been suggested that the dynamic alternations in functional connectivity (FC) may reflect the changes in macroscopic neural activity patterns underlying the critical aspects of cognition and behavior, and thus may act as biomarkers of disease.

**Methods:** In this study, the resting-state functional MRI (fMRI) data were collected from 26 patients with GAD, 22 patients with PD, and 26 healthy controls (HCs). We investigated dynamic functional connectivity (DFC) by using the group spatial independent component analysis, a sliding window approach, and the *k*-means clustering methods. For group comparisons, the temporal properties of DFC states were analyzed statistically.

**Results:** The dynamic analysis demonstrated two discrete connectivity “States” across the entire group, namely, a more segregated State I and a strongly integrated State II. Compared with HCs, patients with both GAD and PD spent more time in the weakly within-network State I, while performing fewer transitions and dwelling shorter in the integrated State II. Additionally, the analysis of DFC strength showed that connections associated with ADs were identified including the regions that belonged to default mode (DM), executive control (EC), and salience (SA) networks, especially the connections between SA and DM networks. However, no significant difference was found between the GAD and PD groups in temporal features and connection strength.

**Conclusions:** More common but less specific alterations were detected in the GAD and PD groups, which implied that they might have similar state-dependent neurophysiological mechanisms and, in addition, could hopefully help us better understand their abnormal affective and cognitive performances in the clinic.

## Introduction

Anxiety disorders (ADs) are a group of mental disorders characterized by excessive fear, anxiety, and related behavioral abnormalities, which have a great impact on the social function and quality of life of the patients, and thus impose a great burden on the family and society (Grupp et al., 2014). Among them, generalized anxiety disorder (GAD) and panic disorder (PD), with anxiety as the core emotional experience, are very similar in clinical features, forms of the disease, and treatment, and often coexist. Specifically, GAD shows a persistent omnidirectional difficulty with autonomic nervous system symptoms, and PD is characterized by a paroxysmal, prominent autonomic symptom, with different degrees of avoidance behavior in both groups. Currently, in order to explore a new diagnostic system in line with the biological findings rather than the clinical symptoms, it is necessary to detect their common and specific neural physiopathology.

Based on the resting-state functional MRI (RS-fMRI) data, the static functional connectivity (FC) analysis has been widely used to indicate the neural physiopathology of various diseases (Bell and Sejnowski, 1995; Lawrie et al., 2002; Greicius et al., 2007; Hahn et al., 2011; Zhong et al., 2018). Nevertheless, different degrees of attention, mind-wandering, and even mood swings may occur during the scanning process, which leads to the observed blood-oxygen-level-dependent (BOLD) signal nonstationary and deviates from the hypothesis. Recently, the temporal features of brain activities, acquired from fMRI, can be characterized by applying a dynamic functional connectivity (DFC) approach (Allen et al., 2014; Calhoun et al., 2014). The full repertoire of the resting-state functional networks has been regarded as continuously and dynamically active (Smith et al., 2009). Current studies have revealed that DFC metrics may index the changes in macroscopic neural activity patterns underlying the critical aspects of cognition and behavior (Hutchison et al., 2013), and thus be of great significance for the early diagnosis and prediction of the severity of mental illness (Damaraju et al., 2014; Li et al., 2014; Ou et al., 2015).

For the brain mechanism of AD, the “limbic-PFC circuitry” was the mainstream view in the past decade. Based on this, Calhoon and Tye (2015) proposed a hypothesis that reconciled the region-specific studies of anxiety into a broader network (Calhoon and Tye, 2015). At present, many studies have progressed in understanding the neural basis of ADs *via* the FC analysis and detected that multiple key functional networks play an important part in generating different symptoms of ADs. For GAD, studies have identified that the amygdala and the prefrontal cortex (PFC) play important roles in “emotion dysregulation” (Hilbert et al., 2014), and that the altered FC was found between these regions of interest (i.e., the amygdala and the PFC) and the default mode network (DMN) (Makovac et al., 2018). Besides, the integrity of functional brain networks was globally disrupted in GAD, showing impairments consistent with the neurobiological models of GAD (involving amygdala, PFC, and cingulate cortex) (Li et al., 2016), appearing that the DMN, cerebellar (CB) network, executive control network (ECN), and salience network (SAN) may be altered (Etkin et al., 2009; Yao et al., 2017). For PD, it is reported that right superior temporal gyrus (STG), left dorsomedial prefrontal cortex (dmPFC), and right orbital frontal cortex (OFC) have a co-atrophy relationship with each other, and these regions are related to the behavioral domains of audition, music, emotion, and execution. The left dorsolateral prefrontal cortex (dlPFC) co-activates with bilateral dmPFC, and these regions are related to the behavioral domains of social cognition and emotion of sadness (Wu et al., 2018). The greater FC between somatosensory cortex and thalamus in PD was more likely linked to interoceptive processing (Cui et al., 2016). Furthermore, the DMN, SAN, and SMN may be altered in PD (Pannekoek et al., 2013; Shin et al., 2013; Kim and Yoon, 2018). In brief, to elucidate diagnostic nosology and promote disorder-specific therapies, it appears to be distinctly necessary for the resting-state studies that directly compare DFC between subjects with different ADs.

In this study, by using the independent component analysis (ICA) and DFC analysis on the RS-fMRI data, we aimed to reveal the characteristics of dynamic connectivity in patients with GAD and with PD. We made the following hypotheses: (1) the patients with AD and healthy controls (HCs) have DFC alterations within the resting-state networks and (2) there are more common and less specific alterations between patients with GAD and PD.

## Materials and Methods

### Participants

Participants were the clinical outpatients consecutively recruited at the Department of Medical Psychology and the Department of Mood Disorders of Nanjing Brain Hospital, affiliated with the Nanjing Medical University. We used the following core inclusion criteria: (1) a primary diagnosis of GAD/PD by an experienced psychiatrist based on the Diagnostic and Statistical Manual of Mental Disorders (Fifth edition, DSM-5TM) (Francesmonneris et al., 2013), (2) a confirmation of GAD/PD diagnosis using Mini-International Neuropsychiatric Interview (MINI), (3) scores ≥14 on the 14-item version of the Hamilton Anxiety Rating Scale (HAMA, Hamilton, 1959), (4) free of psychiatric medications at least 6 months prior to the study enrollment (for GAD only), (5) aged 18–55 years old, and (6) right-handed. The exclusion criteria were as follows: (1) neurological disease, (2) more than one target diagnosis, including psychiatric and personality disorders, (3) severe physical illness, pregnancy, and/or breastfeeding, (4) suicidal risk, (5) inability to complete MRI, and (6) major life change in the last year as defined by death of spouse, unemployment, severe illness, serious injury, legal disputes, property loss, traffic accident, natural disasters, or divorce.

The healthy controls (HCs) matched for gender, age, and education were recruited by the Internet advertisements and posters. We used the following inclusion criteria for HCs: (1) aged 18–55 years old, (2) HAMA (Hamilton, 1959) total score ≤ 7, and (3) right-handed. The exclusion criteria were as follows: (1) comorbid neurological disorders, (2) history of any symptoms consistent with a psychiatric disorder, (3) pregnancy and/or breastfeeding, (4) history of psychological consultation within 3 months of the study enrollment, (5) inability to complete MRI, and (6) major life change in the last year.

### Demographic and Clinical Measures

For all participants, we used a self-report questionnaire to collect their demographic data including gender, age, years of education, handedness, duration of illness (years), history of psychotropic substances, history of psychological counseling, history of physical illness, and so on. Furthermore, the severity of anxiety of each subject was clinically evaluated by using HAMA (Hamilton, 1959).

### MRI Data Acquisition

All the MRI data were obtained using a Siemens 3.0 T scanner at the Department of Radiology, Nanjing Brain Hospital. During fMRI, participants were asked to keep still with their eyes closed and not to think about anything specific or fall asleep. To avoid head movement and noise, foam pads and earplugs were used. T1-weighted anatomical images were obtained using a 3D-GR/IR sequence according to the following scan parameters: matrix = 2562 × 56, field of view (FOV) = 240 × 240 mm, repetition time (TR) = 1,900 ms, echo time (TE) = 2.48 ms, flip angle (FA) = 9°, 176 slices, slice thickness = 1 mm, and spacing between slices = 0 mm. The resting-state data were acquired using the echo-planar imaging sequence according to the following scan parameters: acquisition matrix = 64 × 64, FOV = 240 × 240 mm, TR = 2,000 ms, TE = 30 ms, FA = 90°, 36 slices, slice thickness = 4 mm, and spacing between slices = 4 mm. A total of 250 volumes were recorded in 500 s.

### MRI Data Preprocessing

The preprocessing of RS-fMRI data sets was carried out using REST 1.8 software (http://restfmri.net/forum/) and DPARSF 4.4 software (http://rfmri.org/DPARSF) based on the MATLAB 2019a (version R2019a, MathWorks, Inc., Natick, MA, USA). For each subject, the first 10 volumes of the data set were removed to allow for MR signal equilibrium. The remaining volumes were corrected for the acquisition time delay between slices, realigned to the first volume for head motion correction, specially normalized using the segmented T1-weighted anatomical images, and then smoothed with a 6-mm full-width at half-maximum Gaussian kernel. Participants with head motion exceeding 2 mm or 2° were excluded.

### Group Independent Component Analysis

After the data preprocessing, we analyzed the group spatial ICA by using Group ICA of fMRI Toolbox (GIFT v4.0b) (http://mialab.mrn.org/software/gift/; Calhoun et al., 2002; Erhardt et al., 2011) to decompose the data of all participants into functional networks.

The principal component analysis was used to reduce the dimensionality of data. The subject-specific data were first reduced to 120 independent components (ICs), and then the data were reduced to 100 ICs with the expectation–maximization algorithm (Roweis, 1998) at the group level. We replicated the Infomax ICA algorithm for 20 times (Himberg et al., 2004; Lu et al., 2020) in ICASSO to evaluate the reliability of the decomposition (Bell and Sejnowski, 1995). Then, the subject-specific spatial maps and time courses for each IC were provided using the back-reconstruction approach [group ICA (GICA)] (Calhoun et al., 2001).

**Figure 1:**
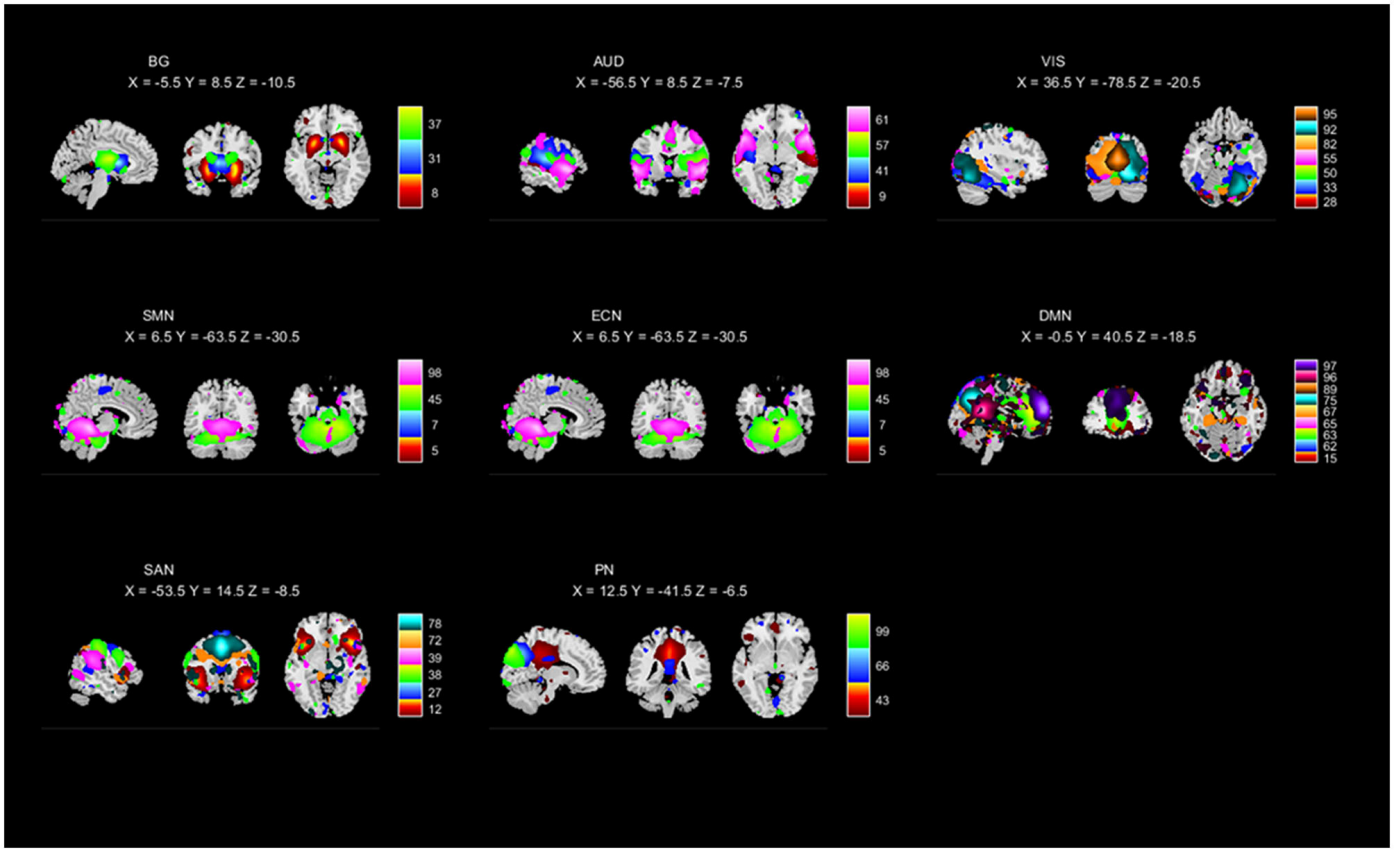
Independent components (ICs; *n* = 42) identified by the group ICA. The IC spatial maps were divided into eight functional networks (namely, BG, AUD, VIS, SMN, ECN, DMN, SAN, and PN).

To perform the selection of ICs, based on the evaluation of the ratio of high- to low-frequency power in the spectra of components as well as whether peak activations took place in gray matter (Robinson et al., 2009; Allen et al., 2011), 42 ICs were identified and categorized into eight functional networks, according to the spatial correlation values between ICs and the templates (http://findlab.stanford.edu/functional_ROIs.html; Shirer et al., 2012; Allen et al., 2014). As shown in Figure 1 and Supplementary Figure 1, the functional networks were arranged into basal ganglia, auditory (AUD), visual (VIS), sensorimotor (SM), executive control (EC), default mode (DM), salience (SA), and precuneus networks. In addition, the subject-specific spatial maps and time courses were post-processed in 3D-DESPIKE (http://afni.nimh.nih.gov/afni), filtering with a high-frequency cutoff of 0.15 Hz.

### Dynamic Functional Connectivity

#### Sliding Window Approach

The sliding window approach is widely used to compute the Pearson's correlation coefficient between time courses of ICs. Following earlier studies, we restricted the window length of 22 TRs with a Gaussian alpha value = 3 and a step of one repetition time (Allen et al., 2014; Damaraju et al., 2014; Kim et al., 2017). Considering that it can be noisy for the covariance estimation, the regularized inverse covariance matrix was used (Varoquaux et al., 2010; Smith et al., 2011). During the whole scan time, 208 consecutive windows were obtained from each subject, in which 42 × 42 pair-wise covariance matrix was calculated. Besides, the L1-norm was imposed to promote sparsity in the graphic LASSO framework with 100 repetitions.

### Clustering Analysis

To assess the frequency and structure of reoccurring FC patterns, as suggested in an earlier study (Allen et al., 2014), the *k*-means clustering algorithm was adopted to cluster all DFC matrices. The L1 distance (i.e., Manhattan distance) function, an effective approach to measure the similarity of high-dimensional data, was used to estimate the similarity between window FC matrices. To determine the optimal number of clusters, we performed the clustering analysis by using the silhouette criterion of the cluster validity index on the subsampling windows of all subjects varying *k* from 2 to 10 (Yao et al., 2019). Finally, *k* = 2 was determined, with the cluster medians regarded as FC states (Supplementary Figure 3). The clustering algorithm was repeated 500 times to increase the chance of escaping the local minima (Wang et al., 2019).

### State Analysis

To investigate the temporal properties of DFC states, we computed fractional windows, mean dwell time, and the number of state transitions for all participants. The “fractional windows” is measured as the number of total windows in each state, the mean “dwell time,” as the average number of consecutive windows in a certain state before switching to another state, and the number of state transitions, as the number of state transitions, which stands for the reliability of each state. Since the effectiveness of the states depended on the span of states in window numbers, we determined the minimum number of windows that a state covered as equal to 10. Specifically, in the approach of the two-sample *t*-test analysis (*p* < 0.05), we examined the group differences in fractional windows, dwell time, and number of transitions, between HCs and patients with AD. The between-group differences among the patients with GAD, patients with PD, and HCs were performed using an ANOVA with age, gender, and years of education as covariates (*p* < 0.05).

Moreover, we performed the two-sample independent *t*-tests to calculate the connectivity strength of each state at each specific regional pairing [i.e., 861 pairings; *p* < 0.05, False Discovery Rate (FDR) correction] between groups.

### Statistical Comparison and Correlation Analysis

For the demographic and clinical characteristics, a permutation one-way ANOVA was used to compare continuous variables, and the chi-square test was used for categorical variables. Additionally, a two-sample *t*-test was performed between the AD subgroups.

For the relationships between altered network temporal properties and clinical variables including HAMA scores and disease duration, we performed the Spearman's correlation analysis in the AD group. All the statistical analyses were performed using SPSS Statistic, release version 26.0 (SPSS, Chicago, IL, USA).

## Results

### Demographic and Clinical Characteristics

Table 1 shows all the statistical differences on the demographic and clinical characteristics among the GAD, PD, and HC groups. No significant differences were found in the age, gender, and education level among the three groups. However, the outcomes of HAMA were significantly different among these three groups at *p* = 0.000.

**Table 1:** Demographic and clinical characteristics of patients with GAD, patients with PD, and HCs in this study.

**Variable**	**GAD (*n* = 26)**	**PD (*n* = 22)**	**HC (*n* = 26)**	***p*-value**
Age (years), mean ± SD	33.75 ± 9.651	35.35 ± 8.715	34.00 ± 9.430	0.817^a^
Sex, males (%)	15(57.69%)	12(54.55%)	13(50.00%)	0.855^b^
Duration of illness (years)	2.214 ± 3.122	2.596 ± 3.180		0.692^c^
Education (years)	12.458 ± 3.605	13.682 ± 3.697	14.846 ± 4.267	0.112^a^
HAMA-T	16.654 ± 5.211	18.500 ± 7.433	2.577 ± 1.758	0.000^a^^*^

*GAD, generalized anxiety disorder; PD, panic disorder; HCs, healthy controls; HAMA-T, the total score of the Hamilton Anxiety Rating Scale*.

a *The p-value was obtained by the permutation ANOVA*.

b *The p-value was obtained by the Pearson's chi-square test*.

c *The p-value was obtained by the two-sample t-test*.

* *Represents the statistically significant value*.

### Intrinsic Connectivity Networks

Based on the anatomical and functional attributes, we grouped all 42 ICs in the following 8 intrinsic connectivity networks: basal ganglia (ICs 8, 31, 37), AUD (ICs 9, 41, 57, 61), VIS (ICs 28, 33, 50, 55, 82, 92, 95), SMN (ICs 5, 7, 45, 98), ECN (ICs 40, 56, 59, 69, 71, 74), DMN (ICs 15, 62, 63, 65, 67, 75, 89, 96, 97), SAN (ICs 12, 27, 38, 39, 72, 78), and PN (ICs 43, 66, 99). The detailed spatial maps of ICs are shown in Figure 1.

### Dynamic Functional Connectivity State Analysis

#### Temporal Properties

**Figure 2:**
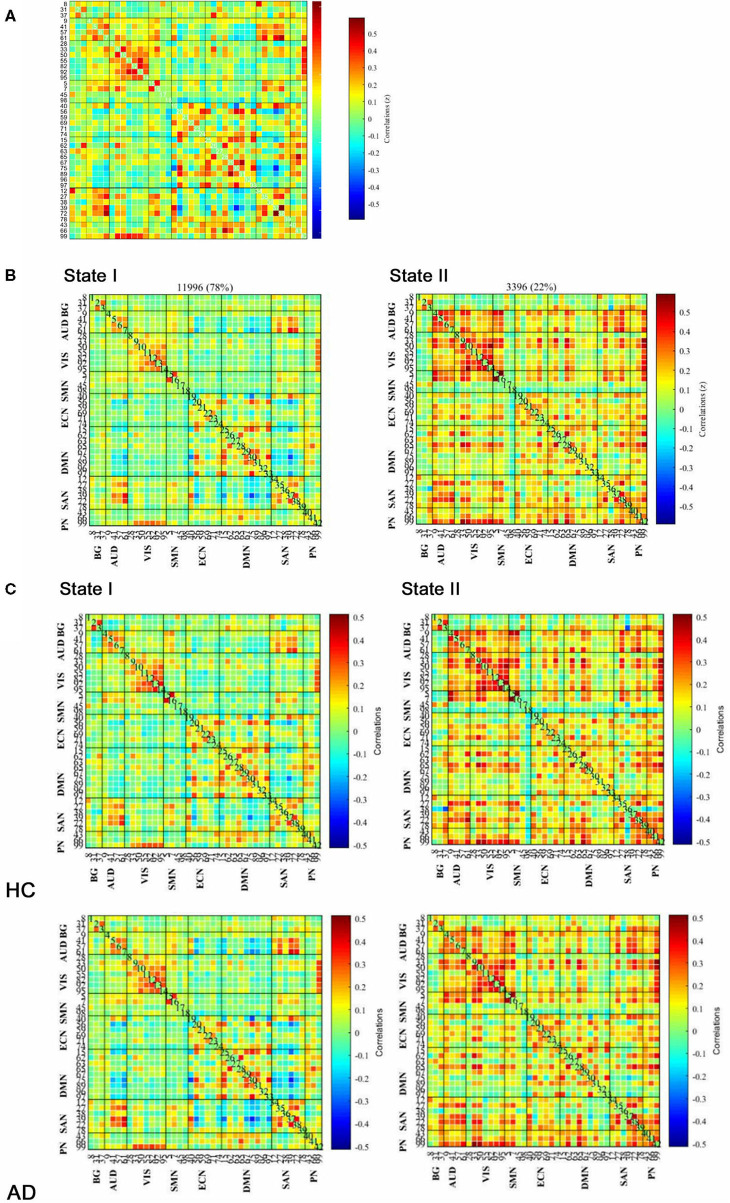
Functional connectivity (FC) results. **(A)** Group-averaged static FC between IC pairs was computed using the entire resting-state data. **(B)** Results of the clustering analysis for each state. The total number of occurrences and the percentage of total occurrences are listed for each state. **(C)** Group centroid matrices and group-averaged across subject-specific median cluster centroids of each group [the percentage of total occurrences for states I and II: 68.20 and 31.80% in the healthy controls (HCs) and 83.21 and 16.79% in the anxiety disorder (AD) groups, respectively]. The value in the correlation matrix represents the Fisher's z-transformed Pearson's correlation coefficient. Based on 8 functional networks, 42 ICs were rearranged individually.

Based on the analysis of all participants, the group average functional connectivity values among the independent components are shown in Figure 2A. For the purpose of the *k*-means clustering method, two patterns of structured FC states were identified which recurred during individual scans and across subjects. As shown in Figure 2B, there are two distinct connectivity “States” across the entire group, namely, a more segregated State I and a strongly integrated State II. Percentages of total occurrences in these two states favored State I (78%) over State II (22%).

As illustrated in Figure 2C, the GAD and PD groups were mentioned as AD group. Group-specific cluster centroids were obtained by the *k*-means clustering algorithm. Generally, regardless of the subpopulation (i.e., HC, GAD, and PD groups), State I contained sparse connections between ICs but exhibited more connections within each intrinsic connectivity network (i.e., AUD, VIS, ECN, DMN, and SAN) as observed through positive coupling. In contrast, State II was noted to own stronger internetwork connections, which involved the AUD, VIS, ECN, DMN, SAN, and PN networks.

**Figure 3:**
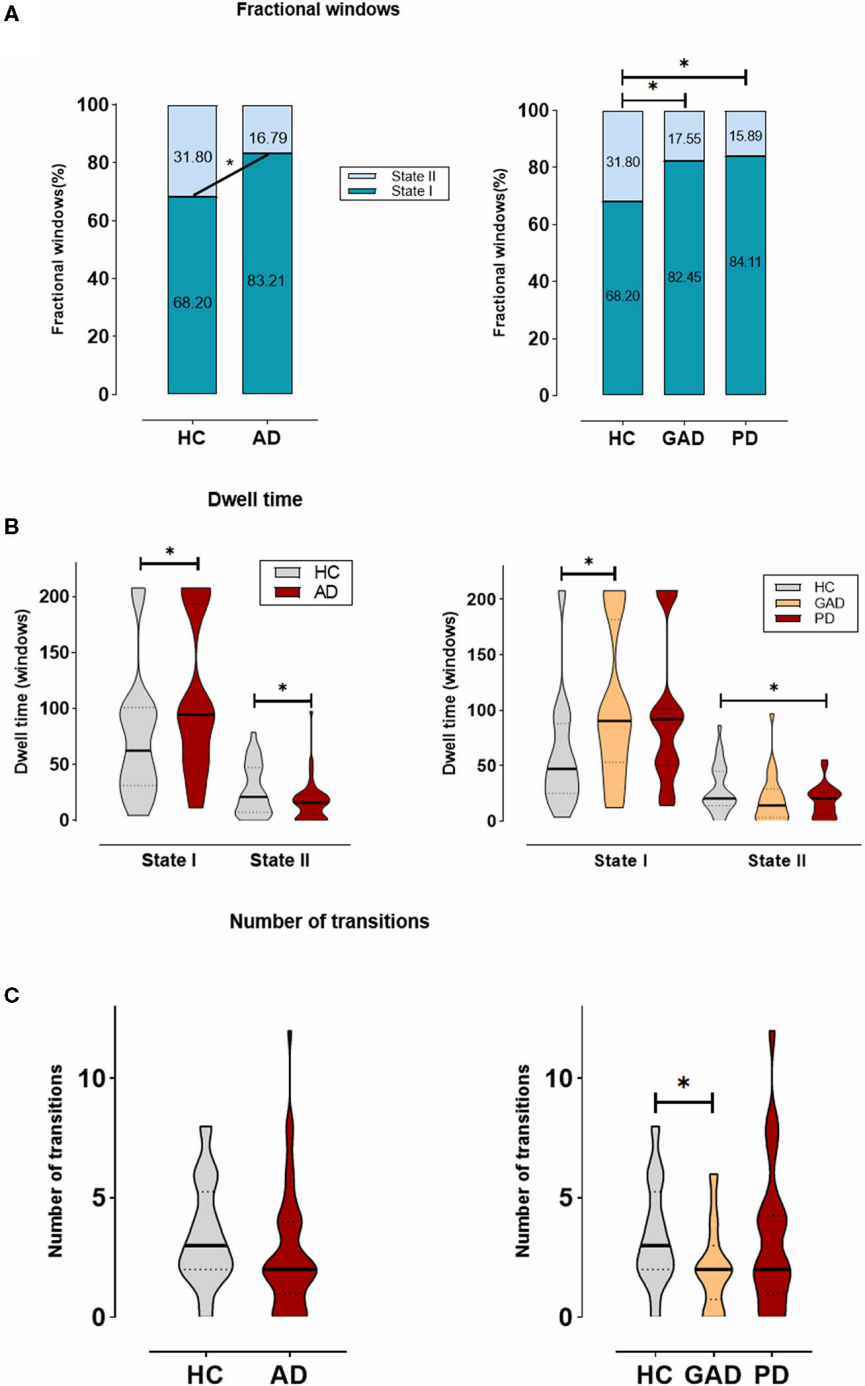
The temporal properties of dynamic functional connectivity (DFC) states between AD and HCs, and also among generalized anxiety disorder (GAD), panic disorder (PD), and HCs. **(A)** The percentage of the mean fractional window subjects spent in each state. **(B)** Mean dwell time. **(C)** Number of transitions were depicted using violin plots. *Indicates that the difference between the two groups is statistically significant (*P* < 0.05).

Besides, there was a significant group difference in fractional windows (*p* < 0.05, two-sample *t*-test). In AD, State I occurred more frequently (83.21 ± 22.20%) than State II (16.79 ± 22.20%). In HCs, comparatively, the total occurrences of State I were observed less frequently (68.20 ± 30.78%; *p* = 0.037) and State II occurred more commonly (31.80 ± 30.78%; *p* = 0.037) by contrast to AD (Figure 3A). In addition, State I in PD (84.11 ± 15.96%; *p* = 0.030) was found significantly different from that in HCs, whereas State I in GAD (82.45 ± 26.32%) was not found significantly different.

Figure 3B shows the significant group differences that were identified in the mean dwell time of each state. Concretely, the mean dwell time of AD group was significantly longer than that of HC group in State I (AD: 109.50 ± 67.90, HCs: 74.67 ± 56.80; *p* = 0.031), whereas the mean dwell time in State II was significantly shorter in the AD group compared with the HC group (AD: 16.86 ± 17.40, HCs: 27.38 ± 22.68; *p* = 0.031). Among the GAD, PD, and HC groups, a further analysis indicated that patients with GAD (116.29 ± 66.93) spent more time in State I than the other groups, while in State II, patients with PD (14.39 ± 9.79) showed a significantly shorter mean dwell time than others.

As shown in Figure 3C, no significant differences were found in regard to the number of transitions between HC group and AD group. Nevertheless, there was a trend for more transitions in the HCs (AD: 2.67 ± 2.50, HCs: 3.42 ± 2.13; *p* = 0.202). Additionally, we compared the AD subgroups to HCs and found the result of a significant difference between GAD and HC groups (GAD: 2.08 ± 1.73; *p* = 0.045).

These changes in the temporal properties showed that patients with AD, including GAD and PD, spend more time in the weakly within-network State I, while performing fewer transitions and dwelling shorter in the integrated State II with the strongly connected functional internetwork components.

### Strength of Dynamic States

**Figure 4:**
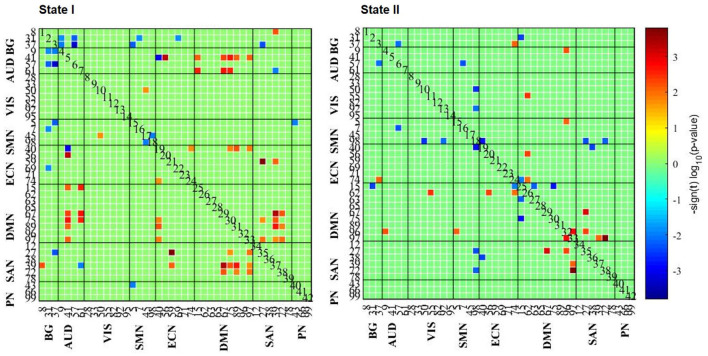
The results of FC strength in each state. The results of the two-sample *t*-test of HC and AD groups are listed, where the AD group had a weaker or stronger FC pattern in comparison with the HCs.

A comparison of the strength of connections among states was performed in the AD and HC groups. As for State I, comparing HCs to AD, we only found three between-network connections (HCs > AD; *p* < 0.05; FDR correction). With the uncorrected condition, there were 26 within- and between-network connections (HCs > AD; *p* < 0.01), 88.46% of which were related to SAN and DMN networks. The remaining connections were in AUD–ECN, VIS–SMN, and ECN–ECN networks. Besides, 12 within- and between-network connections were also found in patients with AD compared with HCs (i.e., BG–AUD, BG–SMN, BG–ECN, BG–SAN, AUD–ECN, AUD–SAN, SMN–SMN, and SMN–PN; AD > HCs; *p* < 0.01; Figure 4).

Comparing HCs to patients with AD, 96.15% (25/26) of connections were in positive relation (*p* < 0.01) to a triple network model related to the aspects of attentive, executive, self-related, and affective information processing domains. However, comparing patients with AD to HCs, 66.67% (8/12) of connections negatively correlated (*p* < 0.01) to BG network. In short, these results indicate that changes in connection strength may reveal the dysfunctional cognitive and psychological processes.

The same analyses for State II were repeated, suggesting that connections were mainly related to DMN network when comparing HCs to patients with AD (10/11) (*p* < 0.01), while connections are stronger involving SMN network when comparing patients with AD to HCs. However, no significant differences were found between GAD and PD groups (*p* < 0.05; FDR correction).

### Relationship With Clinical Properties

As demonstrated in Table 2, the Spearman's analyses were carried out to test the correlations between temporal features and clinical variables in the GAD and PD groups. Specifically, with regard to the GAD group, we observed that the HAMA-sum scores were statistically associated with mean dwell time in each state and fractional windows (*p* < 0.05), and moreover, the HAMA-somatic anxiety scores had an even more significant correlation (*p* < 0.01) with dwell time in State I. Conversely, in the PD group, there turned out to be no significant correlations between time-varying properties and clinical measures. In addition, illness duration seemed to have less relevance to the DFC characteristics.

**Table 2:** The correlations between the temporal properties of dynamic functional connectivity and the clinical characteristics.

**Variable**		**HAMA-T**		**HAMA-S**		**HAMA-P**		**Disease duration**	
		**GAD**	**PD**	**GAD**	**PD**	**GAD**	**PD**	**GAD**	**PD**
Dwell time State I	*r*	0.420	0.185	0.515	0.154	0.263	0.182	−0.343	0.109
	*p*-value	0.032^*^	0.410	0.007^*^	0.494	0.195	0.417	0.093	0.630
Dwell time State II	*r*	−0.440	−0.057	−0.383	−0.059	−0.384	−0.109	0.156	0.052
	*p*-value	0.025^*^	0.801	0.054	0.793	0.053	0.630	0.457	0.818
Fractional windows	*r*	0.471	0.202	0.439	0.178	0.386	0.201	−0.262	0.096
	*p*-value	0.015^*^	0.367	0.025^*^	0.427	0.052	0.370	0.205	0.672
Number of transitions	*r*	−0.358	−0.177	−0.480	−0.157	−0.207	−0.149	0.410	−0.120
	*p*-value	0.073	0.431	0.013^*^	0.485	0.311	0.508	0.042^*^	0.595

*HAMA-T, the total score of the Hamilton Anxiety Rating Scale; HAMA-S, the somatic anxiety score of the Hamilton Anxiety Rating Scale; HAMA-P, the psychic anxiety score of the Hamilton Anxiety Rating Scale*.

* *Represents the statistically significant value*.

## Discussion

In the past few years, many studies have turned to focus on the temporal features in the fluctuations of spontaneous brain activities and come to a preliminary conclusion that DFC may serve as an efficient diagnostic biomarker for neuropsychiatric disease (Allen et al., 2014; Nieuwhof and Helmich, 2017). To our knowledge, this study is the first whole-brain resting-state FC (RSFC) analysis to investigate common and specific time-varying FC alterations between patients with GAD and PD, focusing on the temporal features and the strength of dynamic states.

As stated in this study, two distinct connectivity states were identified across the entire group, one of which was characterized by weakly connected but more frequent, and the other was integrated and strongly connected but less frequent. Comparing patients with AD to HCs, we found that the expression of segregated State I increased by 15.01% in the AD group, while the occurrence of integrated State II turned lower. As concluded in an earlier DFC study on major depression disorder (MDD), the increased occurrence in the weakly connected state indicated that patients with MDD would have more severe depressive symptoms (Yao et al., 2019). By means of the Spearman's correlation analysis, there is significantly positive correlation between fractional windows and HAMA scores, implying that the DFC index may contribute to the diagnosis and the severity evaluation of ADs.

Other temporal properties were also found significantly different among GAD, PD, and HC groups in the segregated state, suggesting that the AD group tends to display a more stable pattern. We observed that the AD group dwelled longer in segregated State I and spent less time in the integrated State II. Compatible with the results obtained in other neurological and psychiatric conditions, the disease group stayed longer in the state with sparse connections (Yao et al., 2016; Liu et al., 2017) and may indicate the decreased information communication (Wang et al., 2019). Furthermore, consistent with evidence suggesting that within-network communication is essential for motor execution, the mean dwell time of the AD group in State I had significant positive correlations with the HAMA-somatic anxiety score. Notably, compared with the PD group, the GAD group occurred more frequently and occupied more time in the segregated State I. Referring to previous findings, it was suggested that the increased FC between hippocampus/parahippocampus and fusiform gyrus in GAD were mainly related to a fear generalization-related neural circuit, which was regarded as evaluation processing, whereas the greater FC between somatosensory cortex and thalamus in PD were more likely correlated to detection processing (Cui et al., 2016). In this respect, our result is in line with what has been reported earlier (Fiorenzato et al., 2019), suggesting that increased brain network functional segregation was closely associated with cognitive performance.

However, contrary to these reports, the reduction in transitioning between two FC patterns fails to distinguish patients with AD from HCs. Meanwhile, among the GAD, PD, and HC groups, the number of transitioning between states in GAD statistically differed from that in HCs, indicating that GAD exhibited more steady dynamic connectivity pattern during the whole scanning. Considering the fact that the rate of transitions may serve as a measure of reduced cognitive flexibility (Guitart-Masip et al., 2016; Yao et al., 2019), our findings suggested that patients with GAD may suffer more severe cognitive dysfunction.

Taken together, our findings show that the DFC properties may efficiently differentiate patients with AD from HCs and thus have the potential of a clinical biomarker. Moreover, further studies with larger sample sizes are needed to confirm whether patients with GAD are more sensitive to the DFC properties than patients with PD.

Further, regarding the strength of dynamic states, we observed that almost half of the within- and between-network connections in two states (26/61) were associated with the triple networks. This model is known as a unifying pattern to explain the neural physiopathology of psychiatric and neurological disorders, involving SAN, DMN, and ECN (Menon, 2011).

Specifically, in the weakly connected State I, the decreased functional connections in ADs were mostly found in SAN–DMN and DMN–ECN. For SAN, it is implicated that the left and right dorsal Anterior Cingulate Cortex (dACC) were involved in the processing of somatosensory information, attentional control, and self-awareness (Bisley and Goldberg, 2010; Koechlin, 2011). For DMN, it is known to be in correlation with self-reference (Kelley et al., 2002; Northoff et al., 2006) and plays an important role in monitoring the internal mental landscape (Greicius et al., 2003; Qin and Northoff, 2011). In particular, many studies have provided clues to FC alterations that both GAD and PD have abnormal RSFC in DMN and SAN (Pannekoek et al., 2013; Andrew et al., 2014; Yao et al., 2017; Kim and Yoon, 2018). Interestingly, we found that the connections between SAN and DMN in ADs were remarkably decreased compared with HCs, suggesting that patients with AD may be weak in identifying the most homeostatically relevant among several internal and external stimuli.

Meanwhile, in accordance with an earlier meta-analysis, decreased connectivity in the DMN and ECN may be related to poor emotion regulation (indicated by hyperactivity of the amygdala), which has been regarded as a central feature in the neuropathophysiology of ADs (Xu et al., 2019). Additionally, the findings in the MDD reported similar results, revealing that the reduced communication in DMN–ECN may be due to difficulty in switching from a “default-state” to an “executive-state” (Hamilton et al., 2013; Mulders et al., 2015; Yao et al., 2019). Notably, for State I, we also observed eight stronger connections between DMN and AUD (HCs > AD), adding to the view that anxiety may lead to poor auditory information processing. Comparing GAD to PD, however, we did not find any significant differences, implying that these two ADs may have common resting-state network activation.

## Limitation

This study should be interpreted cautiously due to several limitations as followed. First, patients with PD were out of drug-free state when the fMRI scanning was performed, so that we cannot exclude the medical effects on FC. Second, taking sample size into consideration, this study may fail to detect some group differences. Despite the reason that the PD group is less sensitive in the DFC analyses, the non-significant values of correlations between DFC features and HAMA scores in the PD group may result from the sample size. Therefore, more participants need to be performed for further verification. Also, according to earlier evidence, the AD is bound up with cognitive and affective dysfunctions (Liberzon et al., 2015; Brinkmann et al., 2017; Neufang et al., 2018), but our brain findings did not correlate with the specific cognitive performance on the attention, executive, and memory domains. Finally, we performed the RS-fMRI data acquisitions in a duration of 8 min for only once, resulting in a lack of two scanning runs for checking the consistency of the analyses. Moreover, comply with an earlier study, the length of resting-state acquisitions needed to be longer than 10 min in the DFC analyses, so as to precisely detect the temporal properties.

## Conclusions

This study analyzed an in-depth assessment regarding DFC features in the GAD and PD groups. Most notably, our findings may suggest that patients with AD can be distinguished from HCs according to the DFC alterations in the resting state. Besides, the GAD group might have the potential to be more sensitive to these time-varying properties than the PD group, which requires further analyses in future studies. Additionally, our study shows that connections associated with ADs were identified including the regions that belonged to DM, EC, and SA networks, especially the connections between SAN and DMN, which may help to explain the abnormal affective and cognitive functions. These findings implied that GAD and PD may have similar state-dependent neurophysiological mechanisms and, in addition, the DFC characteristics could hopefully help us better understand their abnormal affective and cognitive performances in the clinic.

## Data Availability Statement

The original contributions generated for the study are included in the article/Supplementary Material, further inquiries can be directed to the corresponding author/s.

## Ethics Statement

The studies involving human participants were reviewed and approved by Medical ethics committee of Brain Hospital Affiliated to Nanjing Medical University. The patients/participants provided their written informed consent to participate in this study. Written informed consent was obtained from the individual(s) for the publication of any potentially identifiable images or data included in this article.

## Author Contributions

All authors listed have made a substantial, direct and intellectual contribution to the work, and approved it for publication.

## Conflict of Interest

The authors declare that the research was conducted in the absence of any commercial or financial relationships that could be construed as a potential conflict of interest.

## References

[B1] Allen E. A. Damaraju E. Plis S. M. Erhardt E. B. Eichele T. Calhoun V. D. (2014). Tracking whole-brain connectivity dynamics in the resting state. Cereb. Cortex 24, 663–676. 10.1093/cercor/bhs352 23146964 PMC3920766

[B2] Allen E. A. Erhardt E. B. Damaraju E. Gruner W. Segall J. M. Silva R. F.. (2011). A baseline for the multivariate comparison of resting-state networks. Front. Syst. Neurosci. 5:2. 10.3389/fnsys.2011.00002 21442040 PMC3051178

[B3] Andrew P. Paul F. Janine T. Ruth L. (2014). Resting-state neuroimaging studies: a new way of identifying differences and similarities among the anxiety disorders? Can. J. Psychiatry 59, 294–300. 10.1177/070674371405900602 25007403 PMC4079145

[B4] Bell A. J. Sejnowski T. J. (1995). An information-maximization approach to blind separation and blind deconvolution. Neural Comput. 7, 1129–1159. 10.1162/neco.1995.7.6.1129 7584893

[B5] Bisley J. W. Goldberg M. E. (2010). Attention, intention, and priority in the parietal lobe. Annu. Rev. Neurosci. 33, 1–21. 10.1146/annurev-neuro-060909-152823 20192813 PMC3683564

[B6] Brinkmann L. Buff C. Feldker K. Tupak S. V. Becker M. P. I. Herrmann M. J.. (2017). Distinct phasic and sustained brain responses and connectivity of amygdala and bed nucleus of the stria terminalis during threat anticipation in panic disorder. Psychol. Med. 47, 2675–2688. 10.1017/S0033291717001192 28485259

[B7] Calhoon G. G. Tye K. M. (2015). Resolving the neural circuits of anxiety. Nat. Neurosci. 18, 1394–1404. 10.1038/nn.4101 26404714 PMC7575249

[B8] Calhoun V. D. Adali T. Pearlson G. D. Pekar J. J. (2001). Spatial and temporal independent component analysis of functional MRI data containing a pair of task-related waveforms. Hum. Brain Mapp. 13, 43–53. 10.1002/hbm.1024 11284046 PMC6871956

[B9] Calhoun V. D. Adali T. Pearlson G. D. Pekar J. J. (2002). A method for making group inferences from functional MRI data using independent component analysis. Hum. Brain Mapp. 16, 131–131. 10.1002/hbm.10044 11559959 PMC6871952

[B10] Calhoun V. D. Miller R. Pearlson G. Adali T. (2014). The chronnectome: time-varying connectivity networks as the next frontier in fMRI data discovery. Neuron 84, 262–274. 10.1016/j.neuron.2014.10.015 25374354 PMC4372723

[B11] Cui H. Zhang J. Liu Y. Li Q. Li H. Zhang L.. (2016). Differential alterations of resting-state functional connectivity in generalized anxiety disorder and panic disorder. Hum. Brain Mapp. 37, 1459–1473. 10.1002/hbm.23113 26800659 PMC6867341

[B12] Damaraju E. Allen E. A. Belger A. Ford J. M. McEwen S. Mathalon D. H.. (2014). Dynamic functional connectivity analysis reveals transient states of dysconnectivity in schizophrenia. Neuroimage Clin. 5, 298–308. 10.1016/j.nicl.2014.07.003 25161896 PMC4141977

[B13] Erhardt E. B. Rachakonda S. Bedrick E. J. Allen E. A. Adali T. Calhoun V. D. (2011). Comparison of multi-subject ICA methods for analysis of fMRI data. Hum. Brain Mapp. 32, 2075–2095. 10.1002/hbm.21170 21162045 PMC3117074

[B14] Etkin A. Prater K. E. Schatzberg A. F. Menon V. Greicius M. D. (2009). Disrupted amygdalar subregion functional connectivity and evidence of a compensatory network in generalized anxiety disorder. Arch. Gen. Psychiatry 66, 1361–1372. 10.1001/archgenpsychiatry.2009.104 19996041 PMC12553334

[B15] Fiorenzato E. Strafella A. P. Kim J. Schifano R. Weis L. Antonini A.. (2019). Dynamic functional connectivity changes associated with dementia in Parkinson's disease. Brain 142, 2860–2872. 10.1093/brain/awz192 31280293 PMC6736370

[B16] Francesmonneris A. Pincus H. First M. (2013). Diagnostic and Statistical Manual of Mental Disorders: DSM-V. American Psychiatric Association.

[B17] Greicius M. D. Flores B. H. Menon V. Glover G. H. Solvason H. B. Kenna H.. (2007). Resting-state functional connectivity in major depression: abnormally increased contributions from subgenual cingulate cortex and thalamus. Biol. Psychiatry 62, 429–437. 10.1016/j.biopsych.2006.09.020 17210143 PMC2001244

[B18] Greicius M. D. Krasnow B. Reiss A. L. Menon V. (2003). Functional connectivity in the resting brain: a network analysis of the default mode hypothesis. Proc. Natl. Acad. Sci. U.S.A. 100, 253–258. 10.1073/pnas.0135058100 12506194 PMC140943

[B19] Grupp H. Koenig H. H. Konnopka A. (2014). Cost measurement of mental disorders in Germany. J. Ment. Health Policy Econ. 17, 3–8. 24864116

[B20] Guitart-Masip M. Salami A. Garrett D. Rieckmann A. Lindenberger U. Bäckman L. (2016). BOLD variability is related to dopaminergic neurotransmission and cognitive aging. Cereb. Cortex 26, 2074–2083. 10.1093/cercor/bhv029 25750252

[B21] Hahn A. Stein P. Windischberger C. Weissenbacher A. Spindelegger C. Moser E.. (2011). Reduced resting-state functional connectivity between amygdala and orbitofrontal cortex in social anxiety disorder. NeuroImage 56, 881–889. 10.1016/j.neuroimage.2011.02.064 21356318

[B22] Hamilton J. P. Chen M. C. Gotlib I. H. (2013). Neural systems approaches to understanding major depressive disorder: an intrinsic functional organization perspective. Neurobiol. Dis. 52, 4–11. 10.1016/j.nbd.2012.01.015 23477309 PMC3596788

[B23] Hamilton M. (1959). The assessment of anxiety states by rating. Br. J. Med. Psychol. 32, 50–55. 10.1111/j.2044-8341.1959.tb00467.x 13638508

[B24] Hilbert K. Lueken U. Beesdo-Baum K. (2014). Neural structures, functioning and connectivity in Generalized Anxiety Disorder and interaction with neuroendocrine systems: a systematic review. J. Affect. Disord. 158, 114–126. 10.1016/j.jad.2014.01.022 24655775

[B25] Himberg J. Hyvarinen A. Esposito F. (2004). Validating the independent components of neuroimaging time series via clustering and visualization. Neuroimage 22, 1214–1222. 10.1016/j.neuroimage.2004.03.027 15219593

[B26] Hutchison R. M. Womelsdorf T. Allen E. A. Bandettini P. A. Calhoun V. D. Corbetta M.. (2013). Dynamic functional connectivity: promise, issues, and interpretations. Neuroimage 80, 360–378. 10.1016/j.neuroimage.2013.05.079 23707587 PMC3807588

[B27] Kelley W. M. Macrae C. N. Wyland C. L. Caglar S. Inati S. Heatherton T. F. (2002). Finding the self? An event-related fMRI study. Cogn. Neurosci. 14, 785–794. 10.1162/08989290260138672 12167262

[B28] Kim J. Criaud M. Cho S. S. Diez-Cirarda M. Mihaescu A. Coakeley S.. (2017). Abnormal intrinsic brain functional network dynamics in Parkinson's disease. Brain 140, 2955–2967. 10.1093/brain/awx233 29053835 PMC5841202

[B29] Kim Y. K. Yoon H. K. (2018). Common and distinct brain networks underlying panic and social anxiety disorders. Prog. Neuropsychopharmacol. Biol. Psychiatry 80(Pt B), 115–122. 10.1016/j.pnpbp.2017.06.017 28642079

[B30] Koechlin E. (2011). Frontal pole function: what is specifically human? Trends Cogn. Sci. 15:241. 10.1016/j.tics.2011.04.005 21601507

[B31] Lawrie S. M. Buechel C. Whalley H. C. Frith C. D. Friston K. J. Johnstone E. C. (2002). Reduced frontotemporal functional connectivity in schizophrenia associated with auditory hallucinations. Biol. Psychiatry 51, 1008–1011. 10.1016/S0006-3223(02)01316-1 12062886

[B32] Li W. Cui H. Zhu Z. Kong L. Guo Q. Zhu Y.. (2016). Aberrant functional connectivity between the amygdala and the temporal pole in drug-free generalized anxiety disorder. Front. Hum. Neurosci. 10:549. 10.3389/fnhum.2016.00549 27867352 PMC5095112

[B33] Li X. Zhu D. Jiang X. Jin C. Zhang X. Guo L.. (2014). Dynamic functional connectomics signatures for characterization and differentiation of PTSD patients. Hum. Brain Mapp. 35, 1761–1778. 10.1002/hbm.22290 23671011 PMC3928235

[B34] Liberzon I. Duval E. Javanbakht A. (2015). Neural circuits in anxiety and stress disorders: aandnbsp;focused review. Ther. Clin. Risk Manag. 11, 115–126. 10.2147/TCRM.S48528 25670901 PMC4315464

[B35] Liu F. Wang Y. Li M. Wang W. Li R. Zhang Z.. (2017). Dynamic functional network connectivity in idiopathic generalized epilepsy with generalized tonic-clonic seizure. Hum. Brain Mapp. 38, 957–973. 10.1002/hbm.23430 27726245 PMC6866949

[B36] Lu F. Cui Q. Huang X. Li L. Duan X. Chen H.. (2020). Anomalous intrinsic connectivity within and between visual and auditory networks in major depressive disorder. Prog. Neuropsychopharmacol. Biol. Psychiatry 100:109889. 10.1016/j.pnpbp.2020.109889 32067960

[B37] Makovac E. Mancini M. Fagioli S. Watson D. R. Meeten F. Rae C. L.. (2018). Network abnormalities in generalized anxiety pervade beyond the amygdala-pre-frontal cortex circuit: insights from graph theory. Psychiatry Res. Neuroimaging 281, 107–116. 10.1016/j.pscychresns.2018.09.006 30290286

[B38] Menon V. (2011). Large-scale brain networks and psychopathology: a unifying triple network model. Trends Cogn. Sci. 15, 483–506. 10.1016/j.tics.2011.08.003 21908230

[B39] Mulders P. C. van Eijndhoven P. F. Schene A. H. Beckmann C. F. Tendolkar I. (2015). Resting-state functional connectivity in major depressive disorder: a review. Neurosci. Biobehav. Rev. 56, 330–344. 10.1016/j.neubiorev.2015.07.014 26234819

[B40] Neufang S. Geiger M. J. Homola G. A. Mahr M. Schiele M. A. Gehrmann A.. (2018). Cognitive-behavioral therapy effects on alerting network activity and effective connectivity in panic disorder. Eur. Arch. Psychiatry Clin. Neurosci. 269, 587–598. 10.1007/s00406-018-0945-8 30288559

[B41] Nieuwhof F. Helmich R. C. (2017). Entangled cerebral networks in Parkinson's disease. Brain 140, 2767–2769. 10.1093/brain/awx267 29088350

[B42] Northoff G. Heinzel A. de Greck M. Bermpohl F. Dobrowolny H. Panksepp J. (2006). Self-referential processing in our brain-A meta-analysis of imaging studies on the self. Neuroimage 31, 440–457. 10.1016/j.neuroimage.2005.12.002 16466680

[B43] Ou J. Xie L. Jin C. Li X. Zhu D. Jiang R.. (2015). Characterizing and differentiating brain state dynamics via hidden markov models. Brain Topogr. 28, 666–679. 10.1007/s10548-014-0406-2 25331991 PMC4405424

[B44] Pannekoek J. N. Veer I. M. van Tol M. J. van der Werff S. J. Demenescu L. R. Aleman A.. (2013). Aberrant limbic and salience network resting-state functional connectivity in panic disorder without comorbidity. J. Affect. Disord. 145, 29–35. 10.1016/j.jad.2012.07.006 22858265

[B45] Qin P. Northoff G. (2011). How is our self related to midline regions and the default-mode network? Neuroimage 57, 1221–1233. 10.1016/j.neuroimage.2011.05.028 21609772

[B46] Robinson S. Basso G. Soldati N. Sailer U. Jovicich J. Bruzzone L.. (2009). A resting state network in the motor control circuit of the basal ganglia. BMC Neurosci. 10:137. 10.1186/1471-2202-10-137 19930640 PMC2785820

[B47] Roweis S. (1998). EM algorithms for PCA and SPCA. Adv. Neural Inf. Process. Syst. 10, 626–632.

[B48] Shin Y. W. Dzemidzic M. Jo H. J. Long Z. Medlock C. Dydak U.. (2013). Increased resting-state functional connectivity between the anterior cingulate cortex and the precuneus in panic disorder: resting-state connectivity in panic disorder. J. Affect. Disord. 150, 1091–1095. 10.1016/j.jad.2013.04.026 23688914 PMC3759545

[B49] Shirer W. R. Ryali S. Rykhlevskaia E. Menon V. Greicius M. D. (2012). Decoding subject-driven cognitive states with whole-brain connectivity patterns. Cerebral Cortex. 22, 158–165. 10.1093/cercor/bhr099 21616982 PMC3236795

[B50] Smith S. M. Fox P. T. Miller K. L. Glahn D. C. Mickle Fox P. Mackay C. E.. (2009). Correspondence of the brain's functional architecture during activation and rest. Proc. Natl. Acad. Sci. U.S.A. 106, 13040–13045. 10.1073/pnas.0905267106 19620724 PMC2722273

[B51] Smith S. M. Miller K. L. Salimi-Khorshidi G. Webster M. Beckmann C. F. Nichols T. E.. (2011). Network modelling methods for FMRI. Neuroimage 54, 875–891. 10.1016/j.neuroimage.2010.08.063 20817103

[B52] Varoquaux G. Gramfort A. Poline J.-B. Thirion B. (2010). Brain covariance selection: better individual functional connectivity models using population prior, in Paper Presented at the Advances in Neural Information Processing Systems (Vancouver, BC).

[B53] Wang Y. Wang X. Ye L. Yang Q. Cui Q. He Z.. (2019). Spatial complexity of brain signal is altered in patients with generalized anxiety disorder. J. Affect. Disord. 246, 387–393. 10.1016/j.jad.2018.12.107 30597300

[B54] Wu Y. Zhong Y. Ma Z. Lu X. Zhang N. Fox P. T.. (2018). Gray matter changes in panic disorder: a voxel-based meta-analysis and meta-analytic connectivity modeling. Psychiatry Res. Neuroimaging 282, 82–89. 10.1016/j.pscychresns.2018.09.009 30340800

[B55] Xu J. Van Dam N. T. Feng C. Luo Y. Ai H. Gu R.. (2019). Anxious brain networks: a coordinate-based activation likelihood estimation meta-analysis of resting-state functional connectivity studies in anxiety. Neurosci. Biobehav. Rev. 96, 21–30. 10.1016/j.neubiorev.2018.11.005 30452934

[B56] Yao Z. Hu B. Xie Y. Zheng F. Liu G. Chen X.. (2016). Resting-state time-varying analysis reveals aberrant variations of functional connectivity in autism. Front. Hum. Neurosci. 10:463. 10.3389/fnhum.2016.00463 27695408 PMC5025431

[B57] Yao Z. Liao M. Hu T. Zhang Z. Zhao Y. Zheng F.. (2017). An effective method to identify adolescent generalized anxiety disorder by temporal features of dynamic functional connectivity. Front. Hum. Neurosci. 11:492. 10.3389/fnhum.2017.00492 29081741 PMC5645525

[B58] Yao Z. Shi J. Zhang Z. Zheng W. Hu T. Li Y.. (2019). Altered dynamic functional connectivity in weakly-connected state in major depressive disorder. Clin. Neurophysiol. 130, 2096–2104. 10.1016/j.clinph.2019.08.009 31541987

[B59] Zhong Y. Wang C. Gao W. Xiao Q. Lu D. Jiao Q.. (2018). Aberrant resting-state functional connectivity in the default mode network in pediatric bipolar disorder patients with and without psychotic symptoms. Neurosci. Bull. 35, 581–590. 10.1007/s12264-018-0315-6 30515682 PMC6616565

