# Supplementary material for: Intrinsic Network Brain Dysfunction Correlates With Temporal Complexity in Generalized Anxiety Disorder and Panic Disorder
Source: Front Hum Neurosci. 2021 Jul 15;15:647518. doi: 10.3389/fnhum.2021.647518 (PMC8319536; doi:10.3389/fnhum.2021.647518)

**Supplementary Figure 1.** Stacked ortho slices are shown for each selected component in the network. Title shows component numbers plotted from top to bottom.


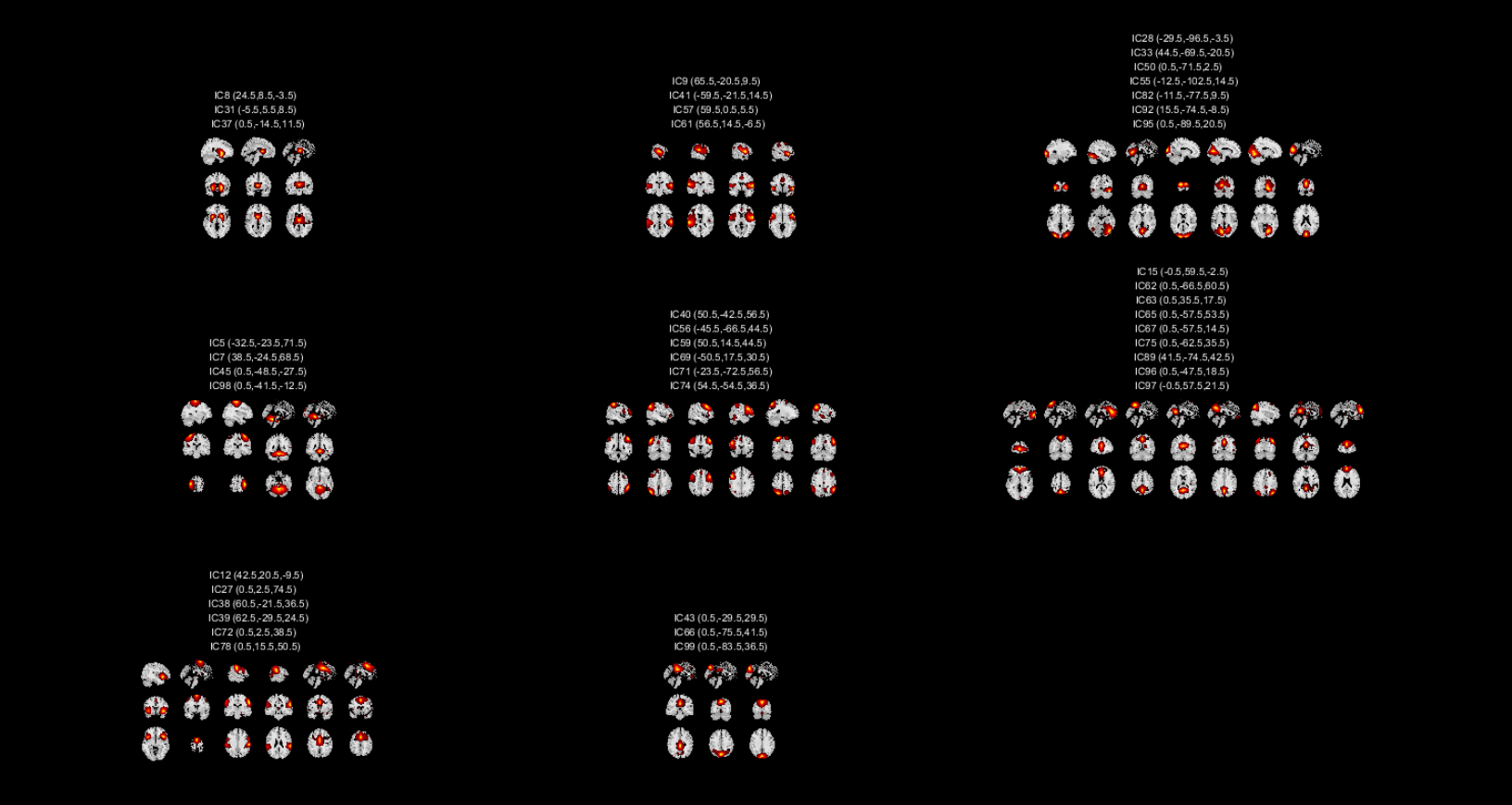


**Supplementary Figure 2.** Stacked ortho slices are shown for each remaining component. Title shows component numbers plotted from top to bottom.


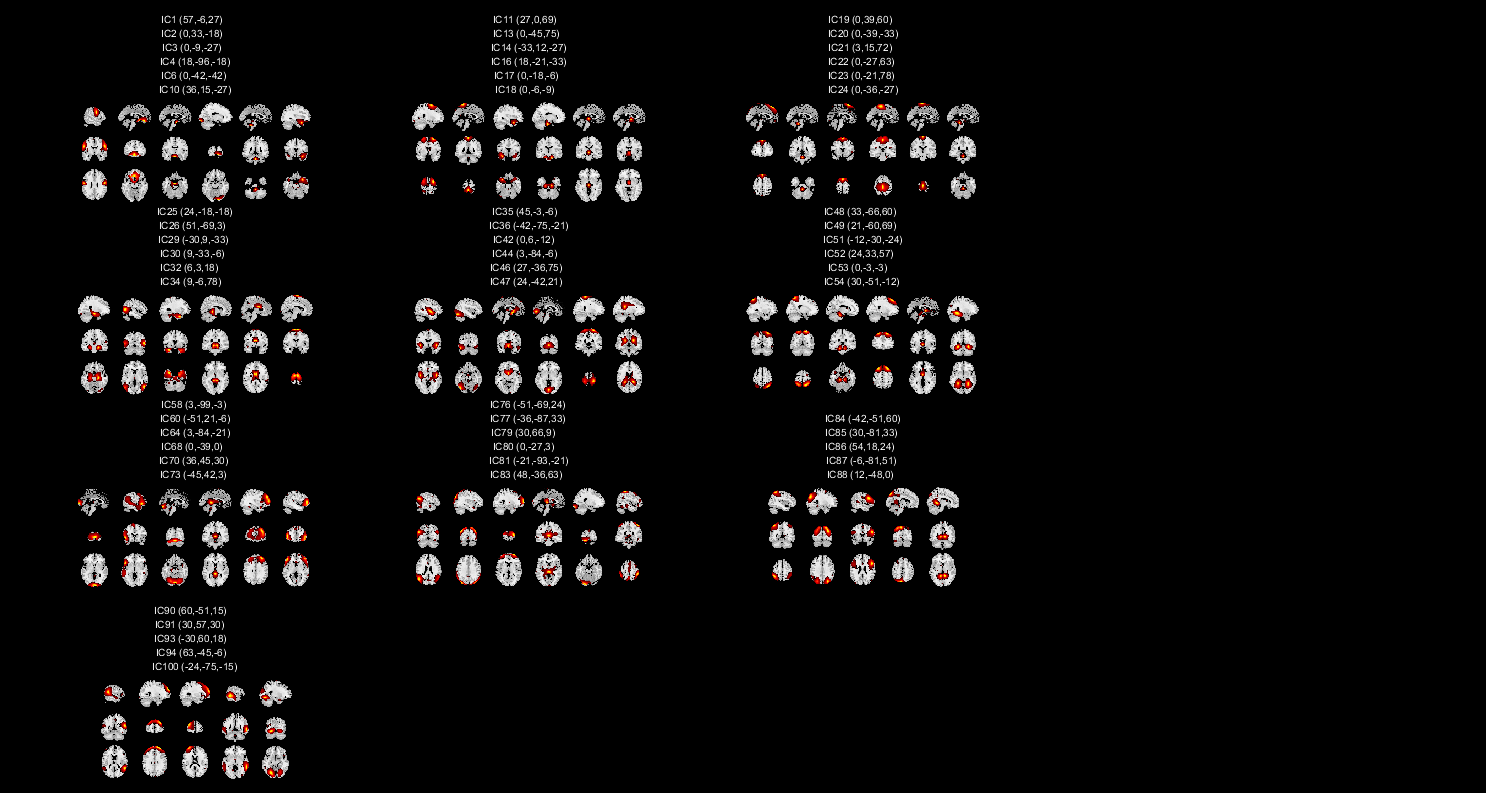


**Supplementary Figure 3.** The silhouette graph to identify the optimal cluster number.


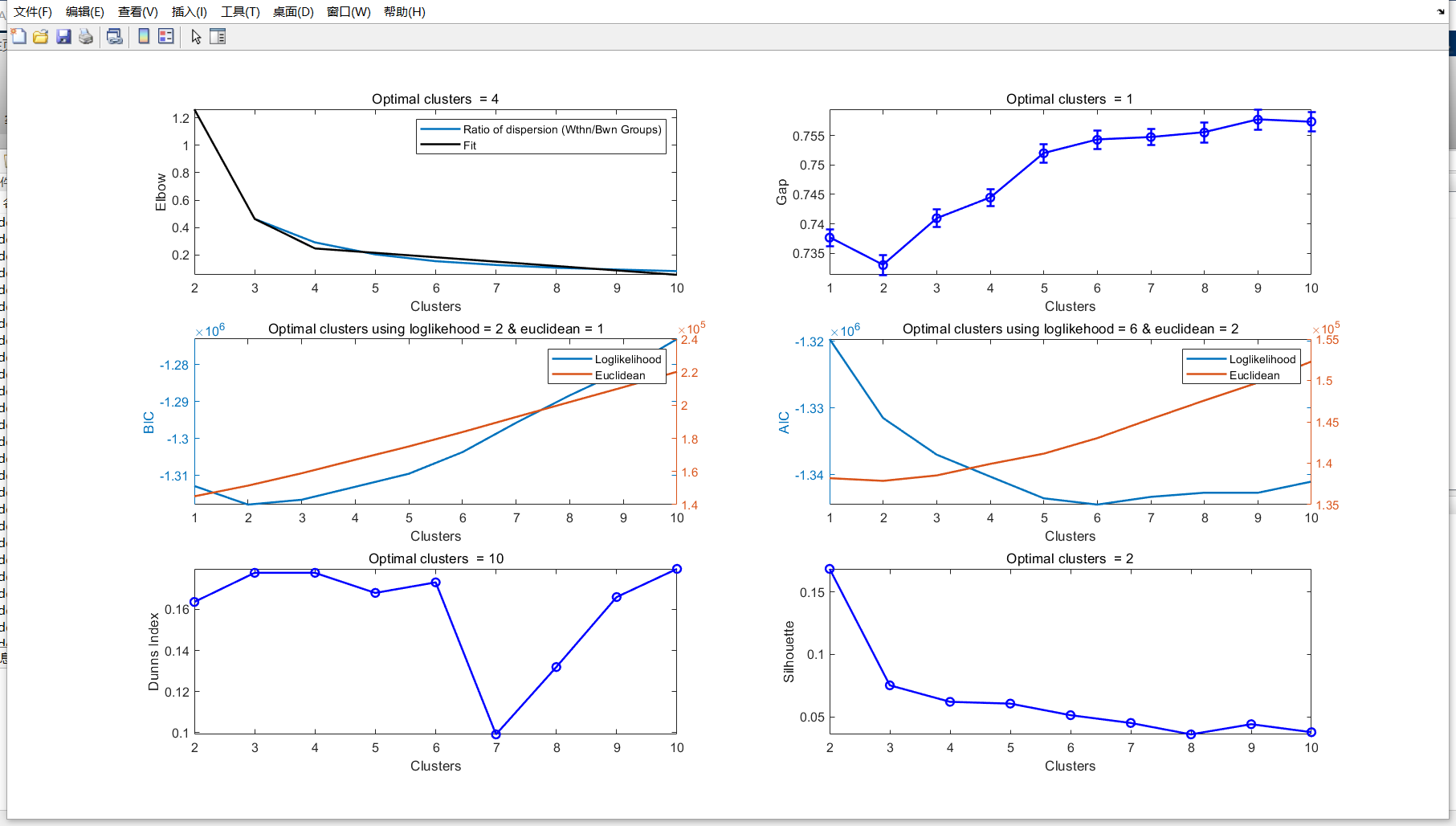

Supplement: Supplementary file 1 [file Data_Sheet_1.docx]
